# The Development of Human Navigation in Middle Childhood: A Narrative Review through Methods, Terminology, and Fundamental Stages

**DOI:** 10.3390/brainsci12081097

**Published:** 2022-08-18

**Authors:** Luca Pullano, Francesca Foti

**Affiliations:** 1 Department of Health Sciences, Magna Graecia University of Catanzaro, 88100 Catanzaro, Italy; 2 Department of Medical and Surgical Sciences, Magna Graecia University of Catanzaro, 88100 Catanzaro, Italy

**Keywords:** navigation, landmarks, spatial cognition, spatial abilities, wayfinding, metric information, physical environment, virtual environment, egocentric, allocentric

## Abstract

Spatial orientation and navigation are fundamental abilities in daily life that develop gradually during childhood, although their development is still not clear. The main aim of the present narrative review was to trace the development of navigational skills in middle childhood (6 to 12 years old) by means of studies present in the literature. To this aim, this review took into account the terminology, methodologies, different paradigms, and apparatuses used to investigate egocentric self-centered and allocentric world-centered representations, besides the different types of spaces (reaching/small/large; physical/virtual). Furthermore, this review provided a brief description of the development of navigational strategies and competences in toddlers and preschool children (0–5 years). The main result of this review showed how middle childhood is a crucial period for the improvement and development of allocentric strategies, including metric information. In fact, during this developmental window, children learn to handle proximal and distal cues, to transpose paper and virtual information into real environments, up to performing similarly to adults. This narrative review could represent a starting point to better clarify the development of navigation and spatial orientation, finalized to trace a development curve useful to map normal development and to have a term of comparison to assess performance in atypical development.

## 1. Introduction

Moving successfully into an environment and reaching set goals is crucial in the daily life of every human being, allowing autonomy and independence [1]. In cognitive terms, this is made possible by spatial navigation and a sense of orientation. In fact, they permit us to organize a path, whether simple or complex, based on two types of representations or frames of reference: egocentric and allocentric. The first refers to body coordinates and expresses the relation of environmental objects with respect to the self [2]; it is mediated by different cognitive and sensory processes such as sight and vestibular and proprioceptive information [3]. The second is more complex to compute and concerns the relationships between the different objects present in the environment. It, therefore, refers to the so-called metric and directional properties of the environments/objects; this involves other cognitive processes such as mental rotation [2]. The acquisition and integration of these two types of representations allow us to generate topographic maps or mental representations of an environment, as first theorized by Tolman [4] and further investigated by O’Keefe and Nadel, who first explored the neurobiological correlates [5].

Importantly, Siegel and White theorized a cognitive model, which, through three progressive and sequential stages, allows the acquisition and organization of environmental information aimed at proper spatial navigation [6]. Specifically, the first stage is the *landmark knowledge*, which usually coincides with the first exposure to a new environment. The peculiarity of this stage is the memorization of objects with relevant characteristics (for example, a building different from the others, which can be referred to as “beacon”), which will then be crucial in the subsequent stages. The second stage is the *route knowledge*, based on egocentric self-centered representations related to previously learned landmarks. The third stage is the *survey knowledge*, based on allocentric world-centered representations, where the metric and directional relationships between the landmarks allow more flexibility in the organization of a path, different from the previously learned one.

According to the Siegel and White model, the transition from one knowledge to another occurs through repeated exposure to an environment, thanks to familiarity. Although this model is currently used and valid, some authors are theorizing new ones.

Namely, Montello has shown how it is possible to compute allocentric representations, i.e., survey knowledge, even from the first exposure to an environment, while familiarity allows us to quantitatively enrich the topographic map that is going to be created [7]. Therefore, this model refutes the sequential processing of environmental information theorized by Siegel and White, indicating that a parallel and simultaneous processing of environmental representations is possible [7].

Recently, real navigational strategies, characterized by a different visual processing, have been considered [8]. In fact, several studies have shown how individuals prefer to adopt one type of strategy over another, which results in differences in terms of performance. One of the most common findings in adulthood is the gender difference in the use of navigational strategies. From this perspective, it was observed that women prefer to use egocentric strategies based on landmarks, reporting lower performance than men, who often use allocentric strategies and metric information [9,10].

The complexity of spatial orientation and the processing of egocentric and allocentric representations are also demonstrated by the neurobiological correlates involved. In fact, a greater involvement of the posterior parietal cortex and the frontal regions has been observed for egocentric representations and encoding of an environment, while the involvement of the hippocampus, parahippocampal area, and retrosplenial cortex has been observed for allocentric representations [11,12,13]. Particularly the retrosplenial cortex seems to be responsible for the integration and switching of egocentric and allocentric representations [10,14].

The development of the neurobiological correlates and, consequently, of spatial orientation seems to be an inverted U-shape, where the highest competence is reached during adulthood [15]. As further evidence, some studies have reported a symmetrical pattern in the use of frames of reference in children aged 6 to 7 years old and elderly aged 80 to 89 years old, characterized by lower accuracy and more spontaneous usage of egocentric representations [15,16]. This parallelism could be related to the immaturity (in the youth) or decay (in the elderly) of the hippocampus, retrosplenial cortex, and frontal areas [15].

Beyond neurobiological correlates, other variables seem to influence the expression of spatial navigation, such as the abovementioned familiarity with the environment [17], spatial anxiety [18], gender differences [1,19], non-verbal memory [20], and even non-spatial factors, such as language [21], making its development difficult to trace. In fact, the evidence is not completely convergent on the stages of spatial navigation acquisition during infancy and childhood [22], although some theories consider childhood as a fundamental age for the emergence of gender differences. Interestingly, according to some theories [1], childhood seems to be crucial for the development of navigational abilities because the time spent outside the house and the distance traveled far from one’s own neighborhood without parents could be related to adult performance and strategies used [1,19]. Specifically, the distance traveled is related to a more frequent use of allocentric strategies and a lower level of spatial anxiety [1,18,23]. This pattern is more specific to males, while females show an opposite pattern with less distance traveled, more anxiety, and more use of egocentric strategies based on landmarks, probably because these are easy to memorize [1]. This could explain the gender differences seen in adulthood, although there are not many longitudinal studies about this topic.

In spite of decades of research on human navigation, the difficulty to trace a clear developmental curve remains, probably due to the complexity of spatial orientation and navigational abilities. Therefore, the aim of the present narrative review was to provide a general overview of the development of navigational abilities in middle childhood (6–12 years) by trying to identify the main stages of its development, namely, summarizing the current knowledge on egocentric and allocentric strategies used, considering that the majority of reviews present in the literature are focused on the precise aspects of human navigation and navigational abilities, without providing a more general view. In this context, the present review can represent a starting point to understand the principal aspects of the development of human navigation. Moreover, while there are some reviews on the age range of 0–5 years (e.g., Newcombe, 2019 [22]; Fernandez-Baizan et al., 2021 [24]), to the best of our knowledge none is present on middle childhood. In considering middle childhood, the present narrative review takes into account some of the main paradigms and methods employed in navigational research over the years as well as the terminology used. Furthermore, this review provides a brief description on the development of navigational abilities/strategies in toddlers and preschool children (0–5 years), useful to understand how the navigational abilities improve and refine during middle childhood.

## 2. Terminology

To better understand the results of the studies discussed in the following sections, terminology will now be introduced in reference to three aspects: landmarks, space size, and type of environment.

*Landmarks*. Landmarks are to be mainly considered of three different types: coincident cue, proximal non-coincident cue, and distal non-coincident cue. For example, let us imagine a doll hidden under a decorated box placed near a toy machine, all of them below a window. The position of the box represents a coincident cue, the toy machine represents a proximal non-coincident cue, and the window could be considered a distal non-coincident cue. Notably, the performances observed in preschool children are also related to how these different landmarks are coded [24].

*Space size*. Another notable difference in spatial navigation and results of the studies concerns the size of the space used. Usually, we can find three types: *large space*, which is at least as large as a gym; *small space*, which is at maximum the size of a room; *reaching space*, which concerns everything at arm’s length, which, therefore, can be manipulated [11]. Another interesting and detailed classification of “psychological space” was made by Montello [25], who defined four types of space: *figural*, *vista*, *environmental*, and *geographical*. The *figural space* defines a fully observable space smaller than the body (such as a picture). The *vista space* is larger than the body but remains fully observable without locomotion (such as a flat plain, such as rooms). The *environmental space* is larger than the body, surrounds it, and requires active locomotion in order to explore, encode, and memorize it. Lastly, the *geographical space* is a space larger than the *environmental space* and it requires the use of maps or models to reduce its size (such as a state, country) [25]. These differences in terms of size are very important because several studies have shown that different navigational strategies and learning methods are involved in the exploration of each space.

*Type of environment.* The last difference concerns the type of the environment where the experimental paradigms are implemented, whether physical or virtual. One of the most debated issues about the type of environment is the lack of real movement and, consequently, the lack of vestibular processes and motor sequences in virtual environments [26]. For this reason, the physical environment has always been considered more reliable and with a higher ecological validity since the same cognitive, sensory, and vestibular processes observed are present during daily life. However, studies with augmented or virtual reality are increasing, supported both by the convenience of design, and by the results that can be comparable to those carried out in a physical environment. Moreover, it has been proven how brain regions and processes involved in physical and virtual environments are similar [27].

## 3. Apparatus/Instruments

In this section, the main apparatus and methods used for the study of navigation in children are described, using the terminology previously introduced.

***Radial Arm Maze (RAM)***. This instrument was designed and used for the first time in 1976 for the study of rodents’ behavior by Olton and Samuelson [28]. The apparatus is formed by a round center with eight arms set at the same distance between them and of the same length. Usually, the paradigm consists of searching for target objects contained in some arms of the maze. To study egocentric representations, the starting point of subjects is always the same, while, to assess allocentric representations, the starting point varies between the training phase and test phase [13]. The RAM falls in the categories of *vista* and *small space* and, depending on the experimental paradigm and *proximal* and *coincident cues*, is usually placed at the entrance or at the end of the arms.

***Morris Water Maze (MWM)***. This instrument was designed and first used by Morris in 1981 to demonstrate that spatial orientation does not require the presence of local cues because, according to him, after several expositions to an environment, it is possible to acquire a topological map [29]. Although the maze is not conventional, the goal is to find a specific target, a hidden platform. In fact, the apparatus is a circular pool filled with opaque water to avoid seeing its depth and the target platform is placed at a certain location. Usually, the circular pool is divided in four quadrants by two bisectors that intersect. The end of each line marks four imaginary cardinal points (north, south, west, and east), which help the experimenters to locate the starting position of the subjects or the location of the platform. Depending on the study, colored shapes could be attached to these cardinal points to help the subjects to orient themselves in the circular pool [30]. This instrument falls into the categories of *vista* and *small space* with, depending on the condition, *distal cues*.

***Kiel Locomotor Maze (KLM)***. This instrument combines elements of RAM and MWM. It was designed by Leplow as one of the first mazes for humans [31]. The apparatus is a circular room, generally 3.6 m in diameter, dimly illuminated, and delimited by black curtains. The only light is provided by a lamp positioned on the edge of the room. The floor of the room is a sort of carpet and, below it, there are 20 LED detectors. The peculiarity of these detectors is the automatic detection of movement above them. In fact, the goal of the KLM is that subjects follow a path indicated by the illuminated detectors. Similar to the MWM, there are two invisible bisectors put at the end of each line as *distal cues*, usually fluorescent foils depicting a sun, a comet, a moon, and stars. Inside the circular room there are two proximal cues, usually a toy mouse and a toy rabbit. Depending on the experiment, the starting position of participants or the rotation of *proximal cues* can change to investigate egocentric or allocentric strategies. This instrument falls into the categories of *vista* and *small space* with *distal* and *proximal cues*.

***Hermer and Spelke paradigm***. This instrument was originally designed by Cheng to study spatial cognition in rats [32] and rearranged by Hermer and Spelke to demonstrate that even toddlers can use a geometric process to re-orient themselves [33,34]. It consists of a simple rectangular room (6.25 × 4.0 × 6.25 ft) with no windows or sources of noise. The room is illuminated by a light put on the center of each wall, while a camera, suspended above the center of the room, records the experiment. The goal of this instrument is to find an object hidden by an experimenter in one of the four corners of the room. Specifically, in the classic paradigm, children are aware of the goal, and they observe the entire scene while the experimenter hides the target object in a corner. After that, children are disoriented by a spinning procedure and then they are asked to find the object. Depending on the experiment, the children’s starting position can change (inside or outside the room), some walls can be of different color to represent *proximal* or *distal cues*, and the shape and size of the room can be different (e.g., triangle, octagon, rhombus), even in the tabletop version. This instrument falls into the categories of *vista* and *small spaces*.

***Nine Box Maze Test Child Version (NBMT-CV)*.** This apparatus was designed by Abrahams and colleagues to investigate spatial memory deficits, based on RAM [35]. There is a children’s version implemented by Pentland and colleagues, aimed to assess verbal and non-verbal aspects of visuo-spatial memory [20]. Particularly, NBMT-CV consists of a square table (74 cm × 74 cm), a series of 10 toys and nine identical cylindrical containers (or “bins”) with detachable lids, four matching chairs placed along the sides of the square table, and seven A4-size photographs of five- and nine-item arrays of the available toys. Furthermore, the task includes three stages: the Object Familiarization, in which the 10 toys are presented to children for 10 s; the Five Box Maze, where five bins are placed in a circle on the table and two toys are hidden inside the bins. After that, all the bins are closed with the lids and the children are asked to move to another chair to disorient them; they are asked to name the hidden toys and indicate the correct bins containing the toys. If the children can recognize all the hidden toys, the A4 photographs with the coincident arrays of toys are shown to help the children to recall the name of the specific array. The last stage is the Nine Box Maze; it is similar to the previous stage but there are nine bins and four hidden toys. The NBMT-CV falls into the categories of *vista* and *reaching spaces* without *cues.*

***View-Independent Point Paradigms.*** These types of paradigms are used to investigate the dependence on a specific point of view to represent an environment. They are an evolution of the well-known Three Mountains Task [36]. Through the years, it was used to study the development of egocentric and allocentric frames of references in toddlers and children. More recently, the paradigms inspired by the Three Mountains Task include a goal where children have to find a hidden object. Examples of this application are the paradigms used by Nardini and colleagues [37,38], where, after being disoriented, children had the goal to retrieve hidden toys from an array of identical “bins” bordered by landmarks or boxes. Usually, these paradigms include three types of frames of reference: the body, the testing room, and the arrays. The disorientation in the children is created by the inconsistency between the frames of references. These instruments fall into the categories of *small spaces* with *proximal cues,* depending on the specific paradigm.

***Star-Maze***. This apparatus is composed by a central pentagon with five alleys radiating from the angles of the pentagon. An example of this maze is reported in one of the most influential studies conducted on this topic by Bullens and colleagues [39]. To measure the allocentric representations, forcing the use of distal cues, the starting point changed. The Star-Maze falls into the categories of *environmental* and *small space* with *distal cues*.

***Cross-Maze***. This instrument consists of a central square with an alley at the center of each of the four sides. Usually, in the egocentric condition, the distance between the starting point and the exit point is always the same; in the allocentric condition, the distance varies; and in the mirror egocentric condition, all the “egocentric” scenarios are inverted [40]. This instrument falls into the categories of *environmental* and *large space* with *distal cues*.

***Regular Maze or Irregular Maze***. These mazes are classical instruments where there are many paths inside a geometric layout such as a square or a rectangle. These types of mazes are used not only to investigate egocentric or allocentric strategies and representations, but also to investigate the “regularity hypothesis” [41]. This hypothesis assumes that the regularity of an environment facilitates the learning of spatial relationships, improving navigation [41]. In fact, these mazes do not present specific cues but walls that can vary their inclination. These mazes fall into the categories of *environmental* and *large space* with *no cues*.

***Hallways***. Another sort of maze is the hallways typology, composed of a series of crossroads where there is only one right choice, while the others are dead ends. Hallways are classically implemented in a virtual environment because of the complexity of geometric layout and size [9,12,42,43]. In terms of the goal, hallways include a starting position and a target location to be reached, such as the standard and classical mazes. At the turn points, the junctions, or at dead ends, there may be *proximal cues* to facilitate orientation. This instrument can fall into the categories of *environmental* and *large* space with or without *proximal cues*.

***Rooms***. This apparatus was used by Hu and colleagues to investigate egocentric and allocentric representations [44]. It consists of a square room (3 m × 3 m, 2.8 m in height). The walls, floor, and ceiling are covered by a gray carpet and, for each wall, there are two LED cubes (80 cm × 80 cm × 80 cm), used as *cues* because their color can be changed by a remote control to investigate allocentric representations. The goal is to find the hidden object after participants have been disoriented. This instrument falls into the categories of *vista* and *small space* with *proximal cues*.

***Reaching space to small space***. Over the years, several apparatuses created for reaching space have been implemented for small space. Particularly, the Four Arm Maze used by Moraleda and colleagues consists of a panel (79 cm × 59 cm) where there are a small, four arm maze (each arm is 30 cm × 3 cm × 2 cm) and five miniature pieces of furniture used as *cues*, while the surrounding room provides *distal cues* [45]. Moreover, based on the classical Corsi Block-tapping Test (CBT) [46], the human-sized Walking Corsi Test (WalCT) [47] and Magic Carpet (MC) [11] were implemented. They are almost the same instruments and follow the same methodology of the CBT; the only difference is the active locomotion required to complete the task. These instruments fall into the categories of *vista* and *small space,* and *cues*, in the traditional form, are not used.

***Memory Island (MI)***. This instrument was developed by Piper and colleagues, and it is a virtual environment (an island) measuring 347 m × 287 m [48] The goal of the participants is to reach a target location positioned in a quadrant. The starting orientation of participants changes in every trial, while the location of the hidden target remains the same. Inside the MI, however, there are several *proximal* and *distal cues* to help the participants’ orientation. This instrument falls into the categories of *environmental* and *large space* with proximal and *distal cues*.

***City***. This instrument was developed by Farran and colleagues and consists of a virtual city (300 × 300 virtual unity), delimited by four walls and containing 10 buildings of different sizes [49]. These are *coincident* or *non-coincident proximal cues*, while outside the three delimitating walls there are three *distal cues*. The starting position is always the same and the nearest wall does not have *cues*. This instrument falls into the categories of *environmental* and *large space* with coincident and *non-coincident proximal cues* and *distal cues*.

## 4. Evidence in Infants and Preschoolers: 0–5 Years Old

The first evidence of spatial navigation starts around 4.5 to 6 months, when children begin to orient themselves using *coincident cues*, but only if they have already explored the environment [24]. Only after 7–8 months of age do they begin to be sensitive to changes, thus starting to use a rudimentary allocentric strategy based on *non-coincident cues*. In fact, at this age, children were able to find hidden objects if the latter remained in the same place [22]. Furthermore, from the age of 18 months, children were capable of orienting themselves efficiently [24] but they only remembered one hidden object [22]. In fact, a rudimentary “path integration”, hence, the ability to track one’s own movement and relocate places and landmarks into an environment, was present starting by the end of the first year, approximately at 12 months [38]. This ability gradually improved up to 2 years of age, at which time the geometric skills necessary for the egocentric and allocentric encoding of the environment began to expand. In fact, several studies using the well-known disorientation paradigm of Hermer and Spelke [33,34] have shown how, starting by the age of 17 months, toddlers were able to reorient themselves and find a hidden object using allocentric features of the environment [50,51,52]. Moreover, it seemed that toddlers were able to use allocentric representations and simple metric information of the environment, for example, the corner of rooms, in different small space shapes, such as rectangles [33,50] and triangles [51,52], even in situations where landmarks were not present. Further evidence, where a similar paradigm was used (i.e., a view-independent point paradigm), showed how, at 3 years of age, children used frame of references based on the room instead of the body, proving the use of allocentric representations [37]. Furthermore, when an inhibition of current strategies based on a precise type of references was required, children aged 3 and 4 failed [37,38], although they began to use shadows as an additional feature of the landmarks in order to orient themselves, allowing a better encoding of *non-coincident* and *proximal cues*. These latter remained the favorite landmarks used for the implementation of navigational strategies, although, at this age, children were also able to use *distal cues* and simple metric information based both on distance among objects in the environment and their length [53]. Moreover, from age 4 onwards, children seemed much less dependent on active movement, orienting themselves just by looking, as usually happens in virtual tasks [54]. Furthermore, at the age of 4, the role of language began to be significant in landmarks’ and objects’ encoding, promoting better navigational performances, especially after children were disoriented [21], although younger children were still less proficient than older children when a major cognitive effort was required [20]. Five-year-old children had no problem using egocentric strategies and they also began to handle allocentric and metric information in a better way [39,55]; moreover, they could switch between egocentric and allocentric strategies and frame of references during a path [37,38,39,44], although with less accuracy than older children [44]. On the other hand, an innovative study has proven how 4- and 5-year-old children could use highly structured strategies in a *vista* and *small space* after a training based on observational learning [56]. Another innovative study conducted by Boccia and colleagues demonstrated how a navigational training administered for 12 weeks improved spatial orientation skills in 4- and 5-year-old children, specifically increasing the transformation of egocentric to allocentric information [57].

Some authors suggested that, as early as 5 and 6 years of age, children were able to encode *proximal* and *distal* landmarks to implement rudimental allocentric strategies in large spaces [39]. Other authors, on the other hand, suggested that this is unlikely before the age of 7 [40]. Probably, these conflicting findings derive from the experimental paradigms employed, which used different types of landmarks and sizes and shapes of spaces. Despite these controversies, everyone agrees on the continuous development and improvement of navigational skills that are fundamental for the use of allocentric representations and strategies [24]. Interestingly, regarding navigational performance, no significant gender differences were found in this age range, as reported by Nazareth and colleagues in their meta-analyses in which they suggested an increase in the gender effect by the age of 13 [19].

## 5. Development of Spatial Abilities in the Range of 6–12 Years

As above mentioned, during the ages 0–5 years, children mainly improve egocentric representations and strategies, although there already are allocentric features, such as the rudimental use of distal landmarks, the use of a view-independent point of view in small and reaching spaces, and the metric and geometric encoding [22,33,37,38,39,50,51,52,53,55]. The age range of 6 to 12 years, as emerged from the studies below and as discussed and reported in Table 1, seems crucial to the development and the improvement of allocentric strategies and the refinement of the already existing egocentric ones.

Particularly, the performance of 6-year-old children was similar in both physical and virtual environments. Children were able to use rudimental allocentric representations in small spaces when proximal cues were present [42,44], but they had serious difficulties when proximal cues were rotated or removed [59]. Furthermore, when landmarks were unavailable, children seemed not capable to use metric and directional information to orient themselves in large spaces [13,42]. Specifically, Yang and colleagues organized the use of metric information in three steps, i.e., individuation of metric information, recognition of their usefulness, and knowledge of how to use this information to locate an environmental goal [13]. Six-year-old children were stuck on the first step. On the other hand, in studies where the Hermer and Spelke disorientation task was used in a simpler and smaller environment, 6-year-old children were capable to use metric and distance information to re-orient themselves [60]. Additionally, in other studies where the view-independent point paradigm was used and the environment was vista and small, 6-year-old children correctly used the different types of frames of reference provided by the environment (egocentric and allocentric) and were able to re-orient themselves correctly [37,38]. Although verbal coding of landmarks helped children to learn the environmental map, thus demonstrating their acquisition of rudimental allocentric representations, they exhibited difficulties in generalizing their knowledge about landmarks, thus encountering difficulties in learning a specific path [63], especially when there were many objects to encode [20]. Furthermore, Lehnung and colleagues proved that 6-year-old children were able to learn a path on a map and then successfully orient themselves when they tested in a real environment [58]. Regarding small space and large space, the children’s favorite strategy was an egocentric one, while an allocentric strategy was used only when they were forced [40]. Eventually, the exploration of 6-year-old children was not organized, as was demonstrated by the fact that they took a lot of pauses, their trajectory was random, and their efficiency was low [49]. In reaching space, the pattern was completely different; in fact, in a study where a tabletop maze was used, 6-year-old children based their performance on allocentric representations of the surrounding room [44,60].

After the age of 5, when children increase their mental rotation ability and the efficiency of allocentric strategies [3], especially in small and reachable spaces [52], the next milestone for the development of navigational abilities is represented by the age of 7, considered a transitional age [39,55,58]. In fact, at the age of 7, the use of allocentric strategies was more stable and spontaneous, although the main strategies used were the egocentric ones, especially in large spaces, where children showed less flexibility in finding short-cuts [65]. Regarding navigational abilities in small spaces, some authors found an allocentric competence similar to adults [44], but with some limitations: for example, children at 7 years of age were capable of using both distal and proximal cues, but when the latter were removed, they tended to be disoriented [55]. Regarding Thorndyke’s “regularity hypothesis” [41], 7-year-old children could not properly use metric information, which is the most challenging aspect of allocentric strategies, and their navigational performance was impaired [61]. The children’s exploration became more organized simultaneously to the development of other cognitive functions such as mental rotation, working memory, non-verbal memory, and word learning as well as spatial memory span [9,11,20,48].

At the age of 8, although the navigational abilities previously developed continue to increase, there is not a leap forward. Therefore, many behaviors of the previous age range are still present, such as errors in navigating both virtual and physical environments, the influence of “regularity hypothesis”, which implies that children cannot properly handle metric information [61], and the inflexible use of allocentric strategies that lead them to a navigational bias when cues are rotated or removed [62]. In a reaching space paradigm, Moraleda and colleagues observed that switching from egocentric to allocentric strategies was still hard because of the failure to inhibit the current strategies used [45]. Nevertheless, children could retrieve a path after a single exposition when proximal or distal cues were present but with more errors and trials taken to reach a goal target compared to adults [43].

The age of 9 represents another milestone for the development of navigational abilities, particularly in the exploration and orienting in large spaces [65]. The spatial abilities were more similar to those of adults and, differently from previous ages, 9-year-old children were capable to find short-cuts, showing an increased flexibility to switch from one strategy to another [65]. Particularly, when landmarks were removed, children used metric information, the last step for improving navigation abilities, although they still committed a few errors in layout recognition based on metric information [13]. According to Farran and colleagues, the exploration of large space by 9-year-old children was very efficient compared to younger children aged 5 to 8, with a lower number of pauses and shorter paths when environments were learned [49].

Starting at 10 years old, through 11 and 12, a constant increase in navigational abilities previously developed was observed; however, in this period there were cognitive differences, such as navigational span memory, that influenced performances [11]. The use of allocentric strategies was more spontaneous, but children still preferred using egocentric ones when possible [39,44]. When the latter was not available, children in this age range were aware of it and they switched to an allocentric strategy without problems [55]. Other sensitive changes included the increased mean speed during exploration of a new environment [39,48,59], the competence to properly use geometric and metric information, the correct orientation when cues were rotated in every type of space [40,45], and the accuracy in learning a new environment after a single exposition [43]. It is astonishing how, at the age of 11, children were able to learn a new environment from a paper map or virtual mode of it and transpose the information in the real environment, showing great allocentric competencies, even when cues were rotated (90° or 180°) or erased, even though there was not any active locomotion during the learning phase [26,65]. Speaking about the differences between children 10, 11, and 12 years old and adults, they can be attributable to brain differences; specifically, children seem to have much more frontal activity than adults, even when motor and attention skills were not required, which translated in a diffuse activation of the brain as well as in a cognitive effort. On the other hand, adults had more functional connectivity in the classical area related to navigational tasks [12]. Finally, speaking about gender differences, as previously said, there was not a clear difference in the navigational performance, although some studies suggested that boys and girls used different cognitive abilities during spatial navigation tasks starting from the age of 6: boys preferred psychometric spatial abilities while girls used more verbal memory during path learning [9], which made them better than boys in locating landmarks, even when the authors did not explicitly invite them to pay attention [64]. These gender differences could be linked to the paradigms used, namely, these differences were found in studies where mental rotation was investigated together with navigational performances [9,64]. Specifically, regarding mental rotation, a recent meta-analysis conducted by Lauer and colleagues [66] showed how, starting from childhood, males showed an advantage in tasks where mental rotation was required. These differences could be fundamental for future differences between males and females, namely, the preference of females to use route strategy based on landmarks [18,67]. Further evidence that supports this theory was the similar performance of 10-year-old girls and adult females, while adult males outperformed 10-year-old boys, demonstrating a continuous development of the boys and a delayed transition in adolescence compared to girls [12]. Thus, it seems that girls reached their navigational potential earlier [12].

## 6. Discussion

Middle childhood is a crucial age for the development of navigational strategies and spatial skills. During the 0–5 age range, children learned to handle rudimental cues, metric information, and allocentric representations, but they mostly used egocentric representations and strategies. Starting from 6 years to 12, children constantly increased their abilities to use allocentric representations and strategies, thus showing a leap forward regarding their navigational competencies. Specifically, by the age of 6, allocentric representations were generated mostly through proximal cues [42,44], although in small, simple environments children were able to use metric and distance information [60]. By the age of 7, children were able to use distal cues, even when cues were rotated, and their competencies were similar to those of adults in small spaces, which represents a milestone in the development of navigational abilities [39,55]. The age of 8 was a stalemate age; however, starting from the age of 9, children’s capabilities grew and expanded to encompass large spaces as well [65]. In fact, metric information was used more frequently than ever. Although children still preferred egocentric strategies, they were able to switch strategies during a task [13]. The ages of 10, 11, and 12 represented the age of refinement for the previously developed and learned abilities: children were capable to use metric information and learn environments from paper maps or virtual environments and apply this knowledge in real life after a single or a few expositions. At this point, navigational span memory, mean speed during navigation, and competence to properly use geometric and metric information and to switch between strategies notably increased, and the performance was adult-like [13,40,45,48]. This improvement in navigational performance was due to a constant brain development and, consequently, to the development and the refinement of underlying cognitive processes such as memory span, attention, encoding, and integration of various types of information, verbal encoding, and mental rotation [9,11,12,48].

No gender differences were revealed in these ages regarding navigational performances, except for a few studies where different strategies were used. These studies showed that boys were more confident with psychometric spatial abilities while girls were more confident with verbal memory [9,64]. Another relevant aspect is the type of environment: interestingly, the results reported in this review came from both the physical and the virtual environment, demonstrating how both types of environments can lead to reliable results and similar conclusions, thus representing the potential of virtual environment paradigms.

In conclusion, the results of the present narrative review shed light on several aspects of the development of human navigation in middle childhood: firstly, the importance of this specific age range in which previously learned navigational skills are refined while new skills are learned and improved; secondly, the diversity of paradigms and methodologies used, although sometimes not permitting a direct comparison of the results from different studies, could represent an advantage allowing a faceted picture of the different navigational abilities and strategies developed during this time.

## Figures and Tables

**Table 1:** Characteristics of studies on spatial abilities in middle childhood.

Study	Group (G): Range Age; Sample Size	Strategies/Measures Assessed	- Test - Physical/Virtual Environment - Type of Space	Main Results
Lehnung et al. (1998) [55]	G 1: 5–5.3; n = 10 G 2: 7–7.3; n = 10 G 3: 10–10.3; n = 10	- Egocentric strategies - Allocentric strategies (NC; P; D)	- Kiel Locomotor Maze - Physical Environment - Small Space	Children at 5 years of age remained bound to an egocentric strategy based on proximal cues and became lost if the proximal cues were removed. Children at 7 years of age seemed to be in a transitional age: five of them demonstrated an egocentric strategy, while the remaining five adopted an allocentric strategy based on distal cues; they had some difficulties when the proximal cues were removed. Children at 10 years of age were able to use allocentric strategies based on distal cues when the egocentric strategies were not efficient, for example, when proximal cues were removed.
Foreman et al. (2000) [26]	G 1: 11; n = 72	- Egocentric strategies - Allocentric strategies (NC; P; D)	- Kiel Locomotor Maze - Physical and Virtual Environment - Small Space	Children at 11 years of age successfully transposed information learned through a virtual environment into a real environment, even in a misleading configuration of Kiel Locomotor Maze in which all objects were rotated 90°. After a few trials, children were able to reach the learning criteria, demonstrating allocentric strategies based on distal cues, even when the proximal cues were rotated 180° or the starting position was changed.
Lehnung et al. (2003) [58]	G 1: 4.3–5.8; n = 48 G 2: 6.4–7.11; n = 48 G 3: 10–12.11; n = 48	- Egocentric strategies - Allocentric strategies (NC; P; D)	- Kiel Locomotor Maze - Physical Environment - Small Space	The peculiarity of this study was its experimental conditions: one group was allowed locomotion inside the maze, while the other group learned the maze by surveying the layout. Children of all age groups successfully transposed information learned by the layout of the environment into real life. Moreover, they were able to orient themselves, although they needed more visits than children of the locomotion group. Note that the children at 5 years of age could only solve the same configuration learned through the layout, following an egocentric strategy. When the proximal cues were rotated, or the starting point was different, 5-year-old children were not capable to use allocentric strategies. Children of 7 years of age assigned to the locomotion group outperformed children that learned the environment through the layout. The performance of 11-year-old children was similar in both conditions.
Leplow et al. (2003) [59]	G 1: 3.1–3.4; n = 16 G 2: 4.1–4.4; n = 16 G 3: 5.1–5.4; n = 16 G 4: 7.1–7.4; n = 16 G 5: 10.1–10.4; n = 16 G 6: 12.1–12.4; n = 16 G 7: 23–25; n = 16	- Egocentric strategies - Allocentric strategies (NC; P; D)	- Kiel Locomotor Maze - Physical Environment - Small Space	No age differences in learning and egocentric phase were observed; number of trials and error scores were proportional with age when locomotion and exploration were allowed. Speed of navigation increased with age. Children below 7 years of age were not able to use the distal cues and failed in the task with rotated proximal cues. Children above 7 years of age were able to master the tasks, and, starting from 7 years old, children used egocentric and allocentric strategies, confirming the transitional age. Particularly, 10- and 12-year-old performances were comparable with adult performance, though the latter were faster and more error free.
Pentland et al. (2003) [20]	G 1: 5–6; n = 20 G 2: 8–9; n = 20 G 3: 11–12; n = 20	- Visuo-spatial memory	- Nine Box Maze Test Child Version - Physical Environment - Reaching Spaces	Children aged 5 and 6 were able to master the Five Box Maze condition of the NBMT-CV, as well as children aged 8, 9, 11, and 12, demonstrating an integration between verbal and non-verbal memory. However, in the Nine Box Maze condition, younger children performed worse than older ones.
Hupbach and Nadel (2005) [60]	*Exp 1.* G 1: 4.0–4.11; n = 16 G 2: 5.6–5.0; n = 16 G 3: 6.6–7.3; n = 14 *Exp. 2* G 1: 2.6–2.11; n = 7 G 2: 3.0–3.11; n = 13 G 3: 4.0–4.11; n = 14 G 4: 5.0–5-10; n = 14 G 5: 6.0–6.11; n = 14	- Allocentric strategies (NC; P; D)	- Hermer and Spelke test, rhombus shape - Physical Environment - Reaching and Small Spaces	In a tabletop apparatus, 4- to 6-year-old children were able to use metric information, specifically angular information, in order to find the hidden object. However, when a relevant landmark was added to the apparatus, children of 4 years of age ignored metric information, while 5- and 6-year-old children combined metric information and landmark information. In small spaces, children younger than 4 years of age searched in a random way; there was no evidence of dominant use of metric or non-metric information as landmarks in children above 4 years of age, although 5- and 6-year-old children performed better.
Jansen-Osmann (2007) [61]	G 1: 7; n = 20 G 2: 11; n = 20 G 3: 24; n = 20	- Regularity Hypothesis	- Ir/Regular Maze - Virtual Environment - Large Space	In a large environment without cues and landmarks, where the wayfinding and spatial knowledge were based on the inclination and angle (for example 135°) of the maze walls, 7- and 8-year-old children seemed to be affected by the irregularity of the walls. Older children and adults had no effects on their performance, demonstrating a wayfinding and a spatial knowledge capable to “regularize” irregular features.
Nardini et al. (2006) [37]	G 1: 3; n = 18 G 2: 4; n = 21 G 3: 5; n = 17 G 4: 6; n = 17	- Spatial frame of references (NC; C; P; D)	- View-Independent point paradigms - Physical Environment - Small Spaces	In a small space where view-independent point paradigms were used, children of 3 years of age were able to use spatial frame of references based on room and body. Interestingly, the use of the room frame was greater than the body one. This preference diminished in other groups over that age range. Furthermore, the array frame was ignored by 3-year-old children, while, starting from 5 years of age, children were able to switch to the appropriate frame of reference to solve the task.
Nardini et al. (2009) [38]	*Exp 1.* G 1: 4; n = 16 G 2: 5; n = 15 G 3: 6–8; n = 18 *Exp 2.* G 1: 5; n = 8 *Exp 3.* G 1: 5; n = 16 *Exp 4.* G 1: 5; n = 13 *Exp 5.* G 1: 5; n = 16	- Spatial frame of references (NC; P)	- View-Independent point paradigms - Physical Environment - Small Spaces	Children at 4 years of age used the same view-dependent strategy in order to retrieve the hidden toys, even when the point of view was changed, thus encoding the visual scene in an elementary way. At 5 years of age, children improved their strategies, but only when movement was allowed. Finally, children aged 6 to 8 were able to re-orient themselves and found the hidden toy in any condition of the study.
Bullens et al. (2010) [39]	G 1: 5; n = 17 G 2: 7; n = 19 G 3: 10; n = 21	- Egocentric strategies - Allocentric strategies (NC; D)	- Star-Maze - Virtual Environment - Small Space	Children at 5 years of age were able to use allocentric strategies when forced to, but rarely they spontaneously used them. Children at 7 years of age more often exhibited spontaneous allocentric strategies than 5-year-old children. Children at 10 years of age used allocentric strategies in a spontaneous way. Furthermore, there was a significant improvement in speed with age. Finally, regarding the mental transformation necessary to create a cognitive map of the environment explored, 10-year-old children outperformed other groups.
Piper et al. (2010) [48]	G 1: 7; n = 12 G 2: 8; n = 15 G 3: 9; n = 11 G 4: 10; n = 12	- Allocentric strategies (NC; C; P) - Memory Span	- Memory Island - Virtual Environment - Large Space	This study did not explicitly assess navigational strategies but spatial memory and its relationship with other cognitive measures. The main outcome was the improvement of spatial memory and mean speed between ages 7 and 10. Particularly, the results evidenced that children with variable attention showed less efficient spatial memory learning and spent less time exploring quadrants of Memory Islands.
Bohbot et al. (2012) [62]	G 1: 8; n = 299 G 2: 21–30; n = 175 G 3: 60–73; n = 112	- Egocentric strategies - Allocentric strategies (NC; P; D)	- Radial-Arm Maze - Virtual Environment - Small Space	Children at 8 years of age more often used spatial strategies, based on landmarks and landscapes, rather than response strategies, based on body-oriented coordinates even when landmarks were erased.
Farran et al. (2012) [63]	G 1: 6; n = 20 G 2: 9; n = 20 G 3 (William Syndrome): 22; n = 14	- Allocentric strategies (P) - Verbal Encoding	- Maze - Virtual Environment - Large Space	The verbal or non-verbal coding impacted on the knowledge of the environment but not on the ability to learn it. Children of 6 years of age had the same accuracy of children of 9 years of age; all children performed better when the cues were easily named.
Moraleda et al. (2013) [45]	*Exp. 1* G 1: 6; n = 24 G 2: 10; n = 24 *Exp. 2* G 1: 6; n = 16 G 2: 8; n = 16 G 3: 10; n = 36 G 4: 19; n = 20	- Egocentric strategies - Allocentric strategies (NC; P; D)	- Four-Arms tabletop - Physical Environment - Reaching Space	Exp. 1: Children of 6 and 10 years of age quickly learned the environment based on guidance cues (cues placed in the same position along the task duration); particularly, 6-year-old children preferred this strategy. In small-scale tabletop. both 6- and 10-year-old children could use allocentric frames of reference based on the configuration of the surrounding room. Children of 10 years of age could use geometric references based on the model, mastering the task when objects were rotated 180°. Exp. 2: Children of 6 years of age could not inhibit their preference for the frames of reference based on the room, while they failed to master the tasks when allocentric frames based on the geometrical and directional characteristics of the model were required. Children of 8 years of age had better performance than 6-year-old children, but they still had problems inhibiting the egocentric and room-based allocentric frames. Lastly, children of 10 years of age performed similarly to young adults; in fact, they could switch between frames of reference and strategies, although they made more errors.
Broadbent et al. (2015) [40]	G 1: 5; n = 16 G 2: 6; n = 15 G 3: 8; n = 17 G 4: 10; n = 16 G 5: (William Syndrome) 21; n = 21	- Egocentric strategies - Allocentric strategies (NC; D)	- Cross Arm Maze - Virtual Environment - Large Space	Children aged 5–10 years spontaneously used egocentric strategies based on the sequence of turns. When the allocentric strategies were the only efficient ones, children aged 5–6 years had difficulties and, in most cases, they failed to complete the task, differently from 8- to 10-year-old children. Finally, in the layout choice (task based on allocentric abilities), the oldest children outperformed those of the 5- and 6-year-old age groups.
Belmonti et al. (2015) [11]	G 1: 6–7.11; n = 23 G 2: 8–9.11; n = 40 G 3: 10–11.11; n = 28 G 4: 21–32; n = 18	- Visuo-spatial memory span	- Magic Carpet - Physical Environment - Small Space	This study did not explicitly assess navigational strategies but the correlation between the spatial memory for navigation and for reaching, as measured by Magic Carpet (MC) and Corsi Block-tapping Test (CBT), respectively. The results evidenced how spatial memory for reaching developed earlier than spatial memory for navigation, but they were correlated. Furthermore, the navigational span increase continued after childhood, as demonstrated by the difference between children aged 10–11 years and adults. No gender differences were revealed in childhood in any of the spans.
Lingwood et al. (2015) [42]	G 1: 6; n = 60 G 2: 8; n = 60 G 3: 10; n = 60 G 4: 20–37; n = 40	- Egocentric strategies - Allocentric strategies (NC; P)	- Maze—Hallways - Virtual Environment - Large Space	Children aged 6 and 8 failed to use a directional (allocentric) strategy to orient themselves in a maze with six junctions, while children of 10 years of age were able to, although they did not perform as well as adults. When landmarks were present inside the maze, the youngest children successfully oriented and completed the task. Finally, verbalizing landmarks provided a better encoding of them, improving environment learning and the replication of paths, although younger children needed more trials than older children and adults.
Merrill et al. (2016) [9]	G 1: 6–12; n = 153	- Allocentric strategies (NC; P)	- Maze—Hallways - Virtual Environment - Large Space	This study investigated the gender differences in wayfinding of children and its relationship with mental rotation, working memory, and word learning. Particularly in boys, there was a significant contribution of psychometric spatial abilities in route learning, in which they performed better than girls. On the other hand, in girls, there was a contribution of verbal memory in route learning performance. According to the model of the authors, the improvement of navigational abilities was not due only to age but also to the development of spatial abilities and verbal memory. Furthermore, small-scale abilities were related to the route learning of children, beginning from 5 and 6 years of age. In route learning performance, differences based on gender became evident at 6 years of age; conversely, none were present in small-scale abilities.
Hu et al. (2018) [44]	G 1: 5; n = 19 G 2: 6; n = 18 G 3: 7; n = 20 G 4: 19–35; n = 53	- Egocentric strategies - Allocentric strategies (NC; C; P)	- Room - Physical Environment - Small Space	Although children aged 5 and 6 were able to use rudimental allocentric representations, they showed less accuracy in the allocentric task compared to children of 7 years of age. Children at 7 years of age showed an allocentric accuracy comparable to adults. Anyway, all groups preferred to use egocentric strategies in every task, but a small number of adults (the most efficient ones) spontaneously used allocentric strategies, even when egocentric strategies could be used.
Lingwood et al. (2018) [43]	G 1: 8; n = 20 G 2: 10; n = 20 G 3: 12; n = 20 G 4: 18–29; n = 20	- Egocentric strategies - Allocentric strategies (NC; P; D)	- Maze—Hallways - Virtual Environment - Large Space	Children aged 8 started to perform similarly to adults, although with more errors and trials. In fact, they could retrieve a new path after a single exposition when landmarks were present. At 10 and 12 years of age, children showed a better performance than younger children, as they learned a new environment after a single exposition.
Murias et al. (2019) [12]	G 1: 10.2–12; n = 15 G 2: 19–34; n = 33	- Allocentric strategies (NC; P; D) - Neurobiological correlates	- Maze—Hallways - Virtual Environment - Large Space	Children of all age groups were not as efficient as adults on the navigational task, employing more time to complete it. Moreover, girls performed similarly to adult females. Notably, the study showed different neural activity between children and adults. Specifically, adults exhibited the classical pattern of areas involved, while children exhibited more frontal activity than adults, probably because they used more motor and attention skills. This evidence demonstrated the effort made by children during navigational tasks and the necessity of the development of the brain to have performance similarly to adults.
Yang et al. (2019) [13]	G 1: 6–8; n = 28 G 2: 9–10; n = 26 G 3: 18–22; n = 27	- Egocentric strategies - Allocentric strategies (NC; P)	- Irregular RAM - Virtual Environment - Large Space	More than half of the children spontaneously used allocentric strategies, while egocentric strategies were rarely used. Nevertheless, children were less efficient than adults in using these strategies. When landmarks were erased, despite children being aware of it, only one-fifth of the 9–10 age group and one-tenth of the 6–8 age group were capable of using metric information as an efficient strategy and not in a spontaneous way, but they switched to it over the course of the trials. Regarding the layout of the environment, 9- and 10-year-old children still had some difficulties in identifying the correct layout, demonstrating the inability to integrate metric information similarly to younger children.
Bocchi et al. (2020) [64]	G 1: 4–10; n = 107	- Egocentric strategies - Allocentric strategies (C; P; D)	- Walking Corsi Test - Physical Environment - Small Space	Girls demonstrated more accuracy than boys in locating landmarks on the map. However, no gender differences were found in the navigational trials or the learning of the sequence of WalCT and its reproduction.
Burles et al. (2020) [65]	G 1: 7; n = 24 G 2: 8; n = 23 G 3: 9; n = 28 G 4: 10; n = 22	- Egocentric strategies - Allocentric strategies (NC; P; D)	- Maze (Museum) - Virtual Environment - Large Space	Children of 7 to 8 years old spontaneously used the previously learned route more than 10-year-old children and adults, demonstrating less flexibility, necessary to find a short-cut. However, at 9 years of age, children seemed to use a proto-cognitive map that permitted them to find short-cuts in an environment, which suggested the age of 9 as a milestone for the development of spatial abilities.
Farran et al. (2022) [49]	Exp. 1 and 2 G 1: 5–11; n = 91	- Egocentric strategies - Allocentric strategies (NC; P; D)	- City - Virtual Environment - Large Space	Exp. 1: There was an increase in explored area of the environment related to the increase in age; this seemed to be the most efficient strategy to learn an environment. Males revisited more places than girls, probably leading to a better performance due to an active exploration, where visiting many areas could contribute to the configural knowledge. Exp. 2: Participants of all age groups reported a linear improvement in navigation success, demonstrating an integration of new objects discovered in every trial, even the younger participants. Interestingly, based on the area explored, pauses taken, and distance traveled, the study proposed three different profiles of explorers: *profile 1* (older male children), characterized by good spatial knowledge of the environment, low number of pauses and revisits, and short path lengths; *profile 2* (older female children and younger children), characterized by limited spatial knowledge, low number of revisits, and high number of pauses; *profile 3* (middle childhood), characterized by average competence, similar but less efficient than profile 1.

C: coincident landmarks; NC: non-coincident landmarks; D: distal landmarks; P: proximal landmarks.

## Data Availability

Not applicable.
